# RABEX-5 Is Upregulated and Plays an Oncogenic Role in Gastric Cancer Development by Activating the VEGF Signaling Pathway

**DOI:** 10.1371/journal.pone.0113891

**Published:** 2014-11-26

**Authors:** Shuang Wang, Aixia Lu, Xiangming Chen, Lin Wei, Jiqiang Ding

**Affiliations:** 1 Department of Clinical Oncology, The Central Hospital of Taian, Taian, Shangdong, People's Republic of China; 2 Department of Nursing, The Central Hospital of Taian, Taian, Shangdong, People's Republic of China; 3 Department of Radiation Oncology, The Cancer Prevention and Control Hospital of Taian, Taian, Shangdong, People's Republic of China; The University of Hong Kong, China

## Abstract

RABEX-5, a guanine-nucleotide exchange factor (GEF) for RAB-5, is implicated in tumorigenesis and in the development of certain human cancers. Here, we report that RABEX-5 promotes tumor growth and the metastatic ability of gastric cancer cells both *in vitro* and *in vivo*. Expression of RABEX-5 is significantly higher in gastric cancer tissues and is associated with tumor size and lymph node metastasis. In addition, targeted silencing of RABEX-5 reduced gastric cancer cell proliferation and colony formation *in vitro* via the induction of a G0/G1 phase arrest, and stimulated gastric cancer cell apoptosis. Knockdown of RABEX-5 also inhibited wound healing, migration and the invasive abilities of gastric cancer cells. The results of *in vivo* animal experiments were also consistent with these *in vitro* findings. Silencing of RABEX-5 led to decreased expression of VEGF. These results indicate that RABEX-5 is upregulated and plays an oncogenic role in gastric cancer development by activating the VEGF signaling pathway.

## Introduction

Despite a decline in the global incidence in certain regions of the world, gastric cancer remains the fourth highest in incidence and second in mortality among all cancers worldwide [1]. Studies indicate that environmental factors, such as *Helicobacter pylori* infection, cigarette smoking and diet, may play an important role in gastric cancer development [2]. Early-stage gastric cancer is usually asymptomatic or associated with nonspecific symptoms, such as dyspepsia, and by the time symptoms occur, it has often reached an advanced stage. Despite the common use of multimodal therapy (chemotherapy, radiation and surgery), the 5-year survival of patients remains low, at 20–40% [3]. Although research in gastric cancer has made great progress, the molecular mechanisms underlying the development of gastric cancer remain incompletely understood.

Exocytosis and endocytosis are key processes used by cells to maintain normal physiological function, and vesicle transport is a core part of this process. Small GTPases play a switch function in intracellular molecules and a very important role in endocytosis and vesicle transport, including the regulation of cell growth, signal transduction, differentiation and actin cytoskeleton building and many aspects of the other cellular processes [4]. In the Ras superfamily, there are more than 50 types of GTPases, including Rab, which belongs to the largest subfamily [5]. Studies have shown that the majority of Rab proteins regulate targeting transport, phagocytosis, and vesicle docking to specific membranes, while a small number of Rab proteins regulate vesicle budding [6], [7]. The small GTPase RAB-5, which is distributed in the plasma membrane and early endosomes, is a master regulator of early endocytic trafficking [8]. Like other small GTPases, RAB-5 is activated by an exchange of bound GDP with GTP, catalyzed by a family of guanine-nucleotide exchange factors [9]. RABEX-5 was identified as an interactor of Rabaptin-5 and possesses GEF activity toward RAB-5 and related GTPases. Likewise, both RABEX-5 and Rabaptin-5 are necessary for RAB-5-driven endosome fusion *in vitro* [10].

Recent studies have indicated that RABEX-5 expression is significantly higher in several cancers, including breast cancer [11], prostate cancer [12] and colorectal cancer [13]. Abnormal RABEX-5 expression in tumors suggests that RABEX-5 is significant in cancer pathogenesis and progression, and may influence tumor biological behavior. However, the role and mechanism of action of RABEX-5 in gastric cancer carcinogenesis and progression have not yet been determined. In this study, we first analyzed expression of RABEX-5 in gastric cancer tissues using quantitative reverse transcription-polymerase chain reaction (qRT-PCR) and immunohistochemistry. Subsequently, we examined the effects of RABEX-5 on tumor biological function *in vitro* and *in vivo*. Our results demonstrate that RABEX-5 is overexpressed and plays an oncogenic role in gastric cancer.

## Materials and Methods

### Ethics Statement

All animal experiments described in this study were approved by the Institutional Animal Care and Use Committee (IACUC) at Taishan Medical University and all animals were maintained in accordance with the IACUC guidelines. The collection and use of human samples were approved by the Institutional Review Board of Taishan Medical University and the affiliated Taian Central Hospital Medical Center, following informed written consent from all patients.

### Patients and tissue specimens

A total of 110 sets of gastric tumors and adjacent, non-tumor tissues (at least 5 cm from the tumor margin) were obtained from patients who underwent curative surgery at Taian Central Hospital between January 2010 and December 2013. These included 27 women and 83 men, with a mean age of 59.3 years (range: 32–80 years). No patients had received radiotherapy or chemotherapy prior to surgery. Clinicopathological data were collected and pathological tumor staging was determined according to the American Joint Committee on Cancer (AJCC) gastric cancer TNM staging system. Histological typing was performed by at least two expert pathologists, working independently in a double-blinded fashion. The study was approved by the Institutional Review Boards of Taishan Medical University and the affiliated Taian Central Hospital Medical Center. Written informed consent was obtained from each patient prior to enrollment in the study.

### Cell lines and culture conditions

Human gastric cancer cell lines and the immortalized gastric, mucosal epithelial cell line, GES-1, were purchased from the Shanghai Institutes for Biological Sciences, Chinese Academy of Sciences. Cell lines were routinely maintained in RPMI-1640 medium containing 10% calf serum, penicillin (100 IU/ml) and streptomycin (100 IU/ml) in a 5% CO_2_ atmosphere at 37°C.

### Total RNA isolation and qRT-PCR

Total RNA was extracted from tissue samples or cell lines using Trizol reagent (Invitrogen, USA), according to the manufacturer's instructions. Reverse transcription was performed using 1 ug of total RNA in accordance with the manufacturer's instructions (Promega, Madison, WI, USA). PCR was performed using SYBR Green reagent (Applied Biosystems, CA, USA) and the following PCR primers; *RABEX-5* (forward) 5′-TTGGACAGATGGAATTGCAA-3′ and (reverse) 5′-GTTGCAGTGGTGGAGGAAGT-3′; *VEGF* (forward) 5′-CTGTACCTCCACCATGCCAAGT-3′ and (reverse) 5′-CTTCGCTGGTAGACATCCATGA-3′ and *GAPDH* (forward) 5′-GGACCTGACCTGCCGTCTAG-3′ and (reverse) 5′-GTAGCCCAGGATGCCCTTGA-3′. PCR reactions were performed with an initial denaturation at 95°C for 2 min, followed by 30 cycles of 94°C for 30 s, 55°C for 30 s and 72°C for 30 s, with a final extension at 72°C for 10 min. *GAPDH* was used as an internal control and all reactions were performed in triplicate. The relative expression ratios of *RABEX-5* in each paired tumor to non-tumor tissue sample were calculated using the 2^−ΔΔCt^ method.

### Immunohistochemistry

Paraffin-embedded tissue sections from patients or nude mice were heat-treated at 60°C for 1 h, dewaxed in xylene, re-hydrated in an ethanol series (100–50%) and treated in 0.01 mol/L citrate buffer (pH 6.0) for antigen retrieval. Endogenous peroxidase activity was inhibited following incubation with methanol containing 0.3% H_2_O_2_ for 30 min. Sections were then incubated with antibodies detecting RABEX-5 (1∶50, Santa Cruz Biotechnology, USA) or VEGF (1∶150; Ab46154, Abcam, USA) at 4°C overnight. The following experimental procedure was incubated with the secondary antibody. Tissue sections were counterstained with Mayer's hematoxylin. Slides were independently evaluated by two pathologists, blinded to any patient data. The staining intensity for RABEX-5 was scored as 0 (no staining), 1 (mild staining), 2 (moderate staining) or 3 (intense staining). Staining area was scored as 0 (0%), 1 (1–25%), 2 (26–50%), 3 (51–75%) or 4 (76–100%), on the basis of the percentage of positively stained cells. The final staining score, calculated as the sum of the intensity and extension scores, was divided into three groups as follows: 0–2, negative expression; 3–4, weak expression; and 5–6, strong expression.

### Targeted silencing of RABEX-5

The RABEX-5 lentiviral interference vector was purchased from Shanghai GenePharma Co., Ltd. The sequence of the RABEX-5 interference targeting oligo was 5′-GGATGCAAACTCGTGGGAA-3′, while the non-homologous control sequence was 5′-TTCTCCGAACGTGTCACGT-3′. SGC-7901 and NCI-N87 cells were plated in 6-well plates (5×10^4^ cells/well), grown to 40% confluence and treated with titered viral supernatant at a multiplicity of infection (m.o.i) of 10.

### Western blot analysis

Whole cell proteins were extracted from cells using RIPA buffer containing Protease Inhibitor Cocktail (Pierce, Rockford, IL, USA). Protein was quantified using a BCA Protein Assay Kit (Pierce). Cell extracts (100 ug) were separated by 10% sodium dodecyl sulfate polyacrylamide gel electrophoresis, and transferred onto polyvinylidene fluoride membranes. Membranes were blocked with 5% non-fat milk in Tris-buffered saline (TBS) and incubated with primary antibodies at 4°C overnight. The primary antibodies used were rabbit anti-RABEX-5 (1∶200; Santa Cruz Biotechnology), rabbit anti-GAPDH (1∶5000; Ab9485, Abcam) and mouse anti-VEGF (1∶300; Ab46154, Abcam). Membranes were then washed three times in TBS-Tween solution for 15 min, and incubated with secondary antibodies. Signals were detected using an enhanced chemiluminescence detection system (Amersham Bioscience, Piscataway, NJ, USA) in accordance with the manufacturer's protocol.

### Cell proliferation assay

Cell proliferation was measured using a Cell Counting Kit-8 (CCK-8; Dojindo Laboratories, Kumamoto, Japan). SGC-7901 or NCI-N87 cells were seeded into 96-well plates (1.5×10^3^ cells/well) and incubated for various timepoints (24, 48, 72, 96 and 120 h). Growth medium was then removed and replaced with 200 µl of RPMI-1640 medium containing 10% FBS and 20 µl of CCK-8 and the plates were incubated at 37°C for 3 h. Absorbance was measured at 450 nm using a Safire2 microplate reader. RPMI-1640 medium containing 10% FBS and CCK-8 was used as a control.

### Colony formation assay

Cells were seeded into 6-well plates (1×10^3^ cells/well) and culture medium was replaced every 3–4 days. After 14 days' culture, cell colonies were fixed with 4% paraformaldehyde for 10 min, stained with 0.1% crystal violet for 20 min, and photographed. Colonies with more than 50 cells were counted in each dash.

### Analysis of cell cycle and apoptosis

SGC-7901/KD, NCI-N87/KD cells and control cells were collected and cell cycle and apoptosis was assessed by flow cytometry. For cell cycle analysis, cells were fixed with 75% ethanol and stored at 4°C overnight. The following day, fixed cells were washed with PBS, treated with RNase A (50 µg/ml) and stained with propidium iodide (PI) (50 µg/ml) for 30 min in the dark. The stained cells were analyzed by flow cytometry (FACSCalibur, Becton-Dickinson).

For apoptosis analysis, an Annexin V-APC Apoptosis Detection Kit I (BD Pharmingen, USA) was used according to the manufacturer's instructions. PI and Annexin V-APC double staining was performed and cells were analyzed by flow cytometry (FACSCalibur, Becton-Dickinson), using the Cell Quest software (Becton-Dickinson). Annexin V-APC-positive and PI-negative cells were defined as undergoing apoptosis. All experiments were performed in triplicate and the average values of these groups were calculated.

### Transwell migration and invasion assay

For migration assays, 1×10^5^ cells were suspended in serum-free RPMI-1640 medium and plated on chambers that were not coated with Matrigel (Corning Costar, NY, USA). For the invasion assay, the upper chamber was precoated with Matrigel (BD Bioscience, CA, USA) according to the manufacturer's protocols, and 1×10^5^ cells in serum-free RPMI-1640 medium were added to the chamber. For both assays, medium containing 10% FBS was added to the lower chamber as a chemoattractant. After 24 h culture, chambers were stained with 0.5% crystal violet solution for 15 min, and immersed in phosphate-buffered saline (PBS) for 10 min. Cells in the lower chamber were subsequently counted under an inverted microscope. Values are expressed as mean cell numbers in five random fields of view (200×).

### Wound-healing assay

Cells were seeded in 6-well plates and cultured until they reached confluence. Wounds were scratched on the monolayer of cells using 20-µL pipette tips. Plates were washed once with fresh medium to remove non-adherent cells following culture of cells for 0 or 48 h (medium without calf serum), and photographed.

### Xenograft model

Four-week-old male BALB/C nude mice were purchased from the Institute of Zoology, Chinese Academy of Sciences of Shanghai. Animals were housed in cages with wood chip beddings in a temperature-controlled room (68–72°F) with a 12-h light-dark cycle and 45–55% relative humidity, and were permitted free access to diet and drinking water. Briefly, SGC-7901/KD or SGC-7901/NC cells were trypsinized and resuspended in PBS (pH 7.4) for injection into one mouse, in a total volume of 100 ul. The cell suspension (1×10^6^ cells) was injected into the right flank of mice. Tumor size was measured externally every 3 days using a caliper, and tumor volume was estimated using the equation: length (mm) × width^2^ (mm) ×0.52. To ameliorate any suffering of mice observed during these experimental studies, mice were euthanized by CO_2_ inhalation on the 28th day after intraperitoneal injection, and tumors were weighed after dissection. Samples were stored in liquid nitrogen for qRT-PCR or fixed in 4% formalin to obtain paraffin-embedded sections. All procedures were conducted according to the Animal Care and Use Guidelines approved by the Taishan Medical University Animal Care Committee.

### Statistical analysis

Data are represented as the mean ± standard deviation (SD). Statistical differences between the two groups were examined by Student's *t*-test. Correlations between RABEX-5 expression in gastric cancer tissues and clinicopathological parameters were analyzed by chi-square or Fisher's exact tests. *P*<0.05 was considered significant, and *P*<0.01 was considered highly significant.

## Results

### RABEX-5 expression is upregulated in gastric cancer tissues

**Figure 1:**
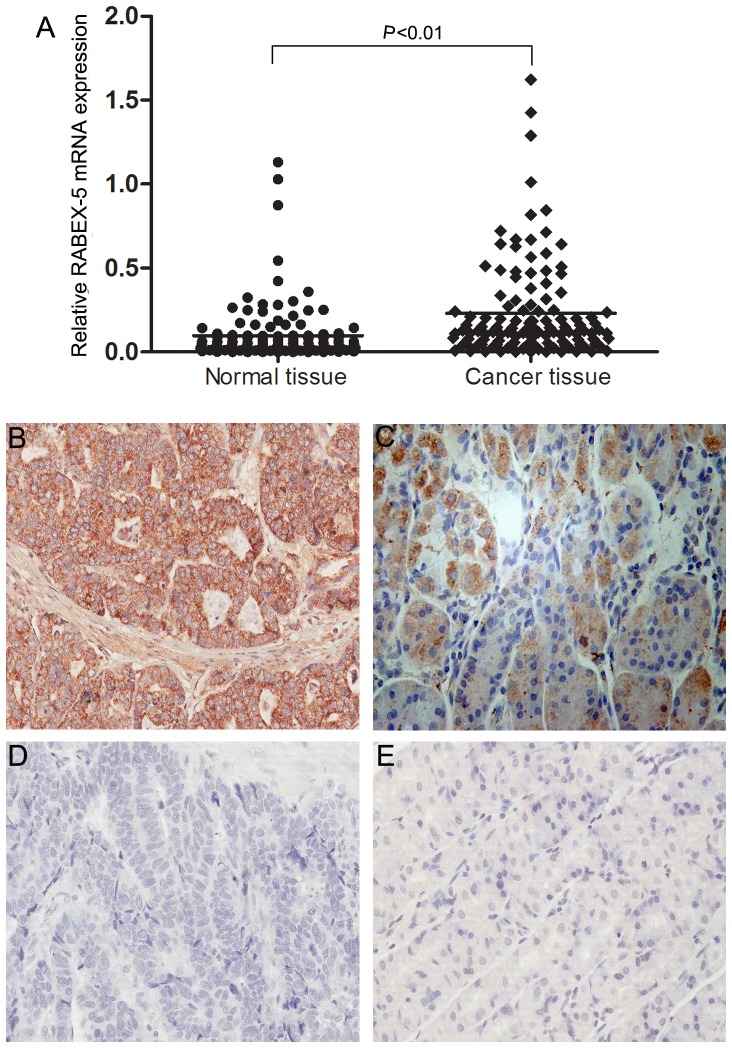
Expression of RABEX-5 in clinical gastric tissues (200×). (A) *RABEX-5* mRNA expression in gastric cancer tissues and paired adjacent non-tumor tissues was examined by quantitative reverse transcription-polymerase chain reaction (qRT-PCR) and normalized to *GAPDH*. Bars represent the means of *RABEX-5* relative expression in cancer tissues and non-tumor tissues, respectively. (B-E) Characterization of RABEX-5 protein expression in human gastric cancer tissues and paired adjacent non-tumor tissues by immunohistochemical staining. (B) Strong positive RABEX-5 expression in gastric cancer. (C) Weak positive RABEX-5 expression in gastric cancer. (D) Negative RABEX-5 expression in gastric cancer. (E) Negative RABEX-5 expression in non-tumor gastric mucosa.

The expression of *RABEX-5* was examined in gastric cancer and adjacent non-tumor tissues by qRT-PCR. We observed significantly higher expression of *RABEX-5* mRNA in gastric cancer tissues compared with corresponding non-tumor tissues (*P*<0.01, Fig. 1A). These results were confirmed at the protein level by immunohistochemical staining (Fig. 1B, C, D, E). RABEX-5 expression was predominantly localized in the cytoplasm of cancer cells. RABEX-5 was upregulated in 61.8% (68/110) of gastric cancer patients. We next investigated the relationship between RABEX-5 expression and clinicopathologic features of gastric cancer. Upregulation of RABEX-5 was associated with tumor size (*P* = 0.035) and lymph node metastasis (*P* = 0.006), but not with other clinicopathological factors including sex, age or tumor location (Table 1).

**Table 1:** Association between RABEX-5 expression and clinicopathological factors of gastric cancer patients.

Variables	Number of cases	RABEX-5 immunostaining		*P*
		Positive(n = 68)	Negative(n = 42)	
Gender				
Male	82	49	33	0.446
Female	28	19	9	
Age(years)				
≥60	70	40	30	0.182
<60	40	28	12	
Tumor differentiation				
Well to moderate	44	26	18	0.631
Poor	66	42	24	
Tumor location				
Gastric fundus	5	2	3	0.568
Gastric corpus	53	34	19	
Pylorus	52	32	20	
Tumor size				
≤3cm	54	28	26	0.035
>3cm	56	40	16	
T stage				
T1+T2	46	32	14	0.156
T3+T4	64	36	28	
Lymph node metastasis				
Negative	45	21	24	0.006
Positive	65	47	18	
Distant metastasis				
Negative	106	64	42	0.109
Positive	4	4	0	
TNM stage				
I+II	42	23	19	0.231
III+IV	68	45	23	

Note: Positive RABEX-5 expression included all positive cases, such as weak and strong.

### Downregulation of RABEX-5 inhibits gastric cancer cell proliferation and colony formation

**Figure 2:**
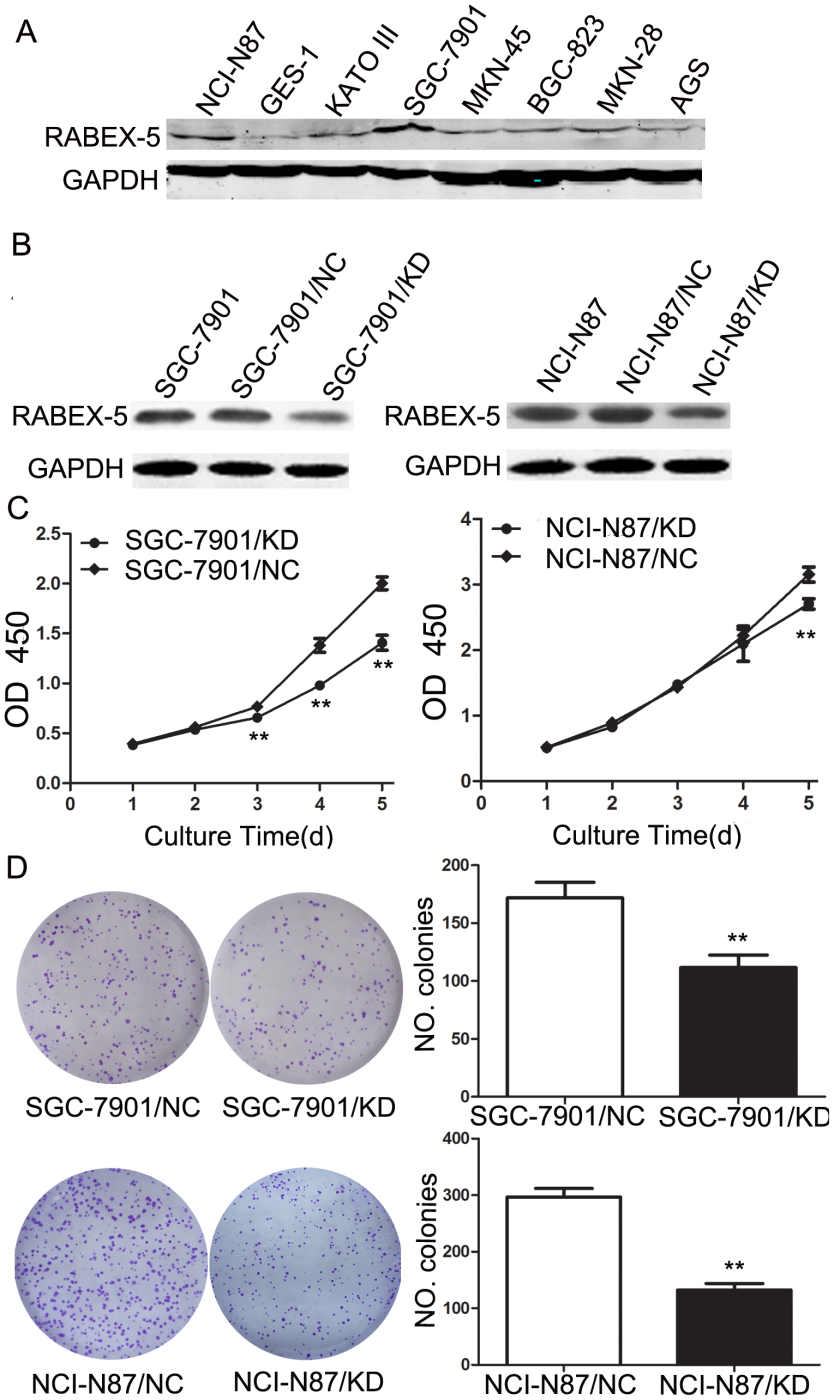
Downregulation of RABEX-5 in SGC-7901 and NCI-N87 cells and effects on cell proliferation and colony formation. (A) Western blot analysis of RABEX-5 protein expression in seven gastric cancer cell lines and GES-1. (B) RABEX-5 protein levels in SGC-7901/KD, SGC-7901/NC, NCI-N87/KD and NCI-N87/NC cells were analyzed by western blot. (C) CCK-8 cell proliferation assay for SGC-7901/KD, NCI-N87/KD and control groups. Curves indicate a significant level of proliferation compared with controls (*P*<0.05). (D) Representative images and quantification of colony formation assays in each cell line. *P<0.05; **P<0.01.

We next investigated the expression of RABEX-5 in gastric cell lines and the non-cancer cell line, GES-1, by western blot analysis. RABEX-5 was expressed in all cell lines tested, and was especially high in SGC-7901 and NCI-N87 cells (Fig. 2A). To explore the functions of RABEX-5 in gastric cancer cell lines, we performed targeted knockdown of RABEX-5 in the high-expressing cell lines, SGC-7901 and NCI-N87. RABEX-5 expression was significantly decreased in SGC-7901/KD and NCI-N87/KD cells compared with control cells (Fig. 2B). First, we examined the effect of loss of RABEX-5 on the growth of gastric cancer cells. After 5 days of culture, the growth rate of SGC-7901/KD and NCI-N87/KD cells was significantly slower than control groups (Fig. 2C). We also confirmed these observations in colony formation assays. Colony numbers were significantly reduced in SGC-7901/KD cells and NCI-N87/KD cells compared with control cells (Fig. 2D), and we observed that colony size was also smaller in experimental groups compared with controls. These data suggest that downregulation of RABEX-5 suppresses gastric cancer cell proliferation.

### Downregulation of RABEX-5 promotes apoptosis of gastric cancer cells and induces G0/G1 cell cycle arrest

**Figure 3:**
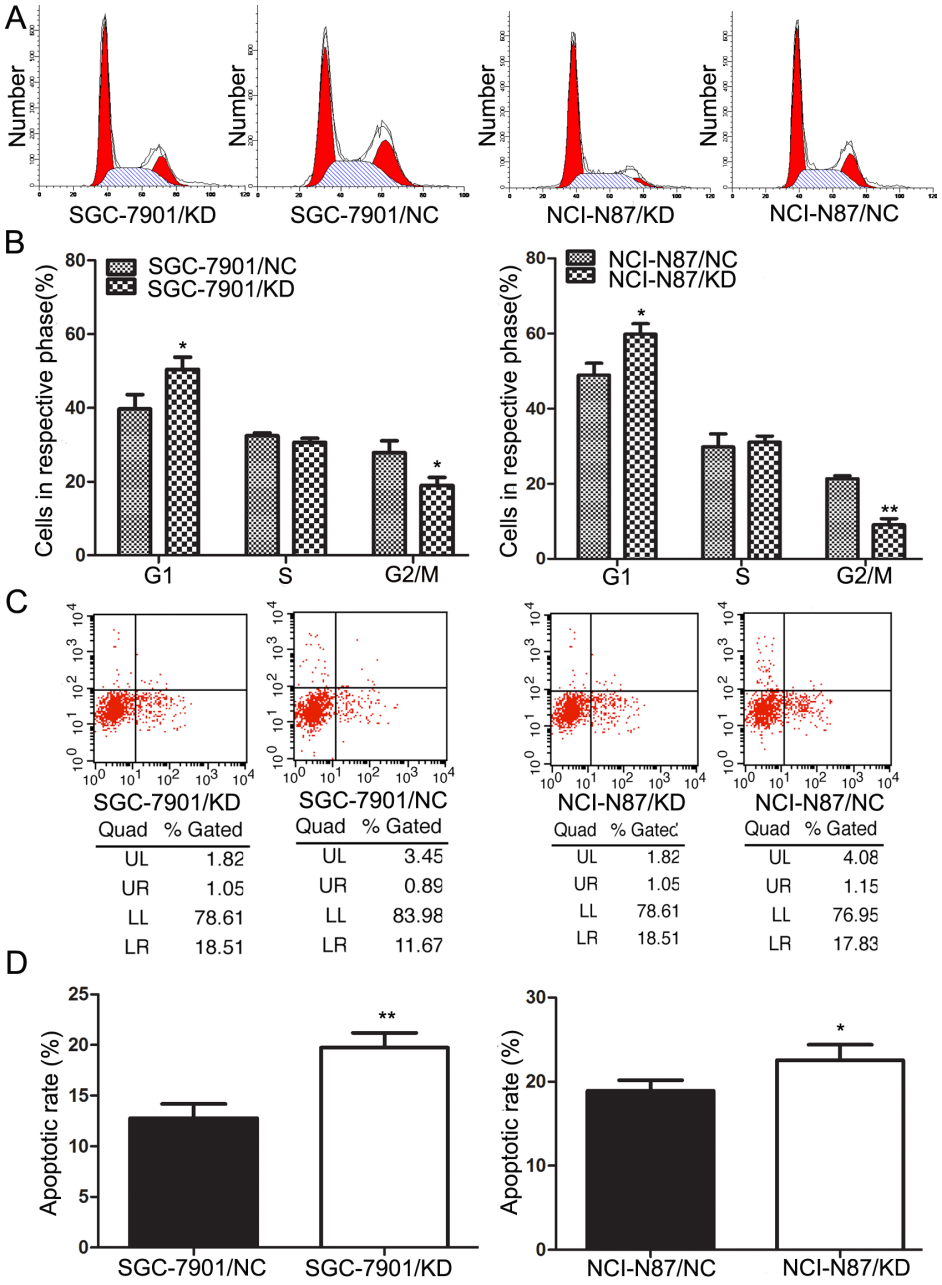
The effect of RABEX-5 on cell cycle distribution and apoptosis of gastric cancer cells. (A) Proportion of cells in various phases of the cell cycle. (B) Representative histograms depicting cell cycle profiles of SGC-7901/KD, SGC-7901/NC, NCI-N87/KD and NCI-N87/NC cells. (C) Cells staining positive for Annexin V-APC and negative for propidium iodide (PI) were considered to have undergone apoptosis. (D) Representative histograms depicting apoptosis of each group of gastric cancer cells. *P<0.05; **P<0.01.

The observed effects of RABEX-5 knockdown on cellular growth may be due to changes in cell cycle progression and apoptosis. We therefore examined the effects of RABEX-5 silencing on cell cycle and apoptosis *in vitro*, using flow cytometry. Flow cytometric analysis revealed that the proportion of cells in the G0/G1 phase in SGC-7901/KD or NCI-N87/KD cells was higher than in SGC-7901/NC or NCI-N87/NC control groups, while the percentage of cells in the G2/M phase was significantly decreased compared with controls (Fig. 3A, B). We also observed significant effects on apoptosis in the differently treated groups (Fig. 3C, D), with downregulation of RABEX-5 expression leading to an increase in gastric cancer cell apoptosis.

### Downregulation of RABEX-5 inhibits migration, invasion and wound-healing ability of gastric cancer cells

**Figure 4:**
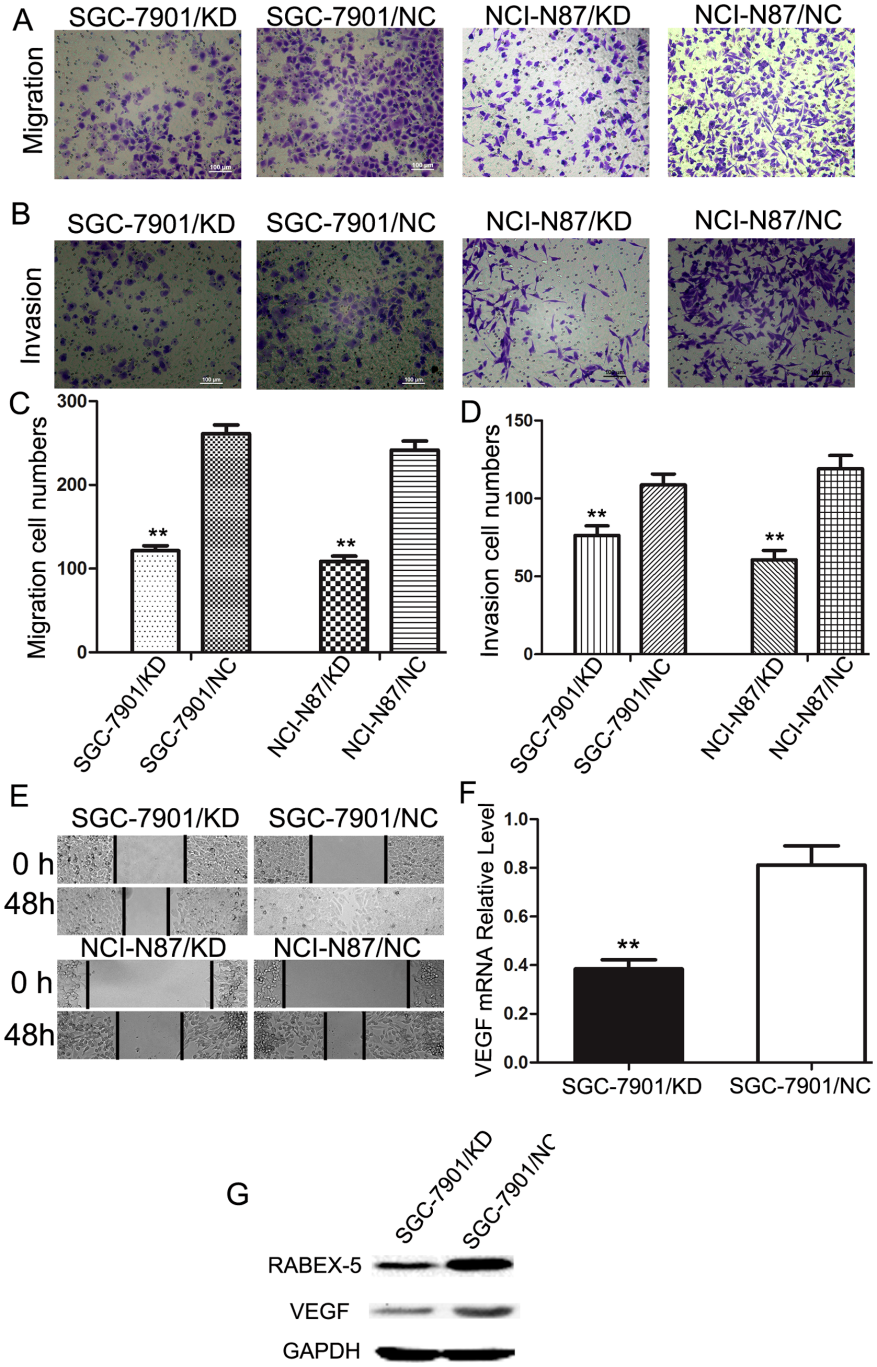
Downregulation of RABEX-5 expression inhibits cell migration and invasion. (A, B) Transwell assays. Photographs showing cells following migration through micropore membranes with or without Matrigel. (C, D) Histograms depicting the numbers of migrating and invading cells. (E) Wound-healing assay with gastric cancer cells. Images were taken 0 and 48 h after scratching the cell surface. A representative image from three independent experiments is shown. (F) *VEGF* mRNA expression was evaluated by qRT-PCR. (G) VEGF protein expression was evaluated by western blot. *P<0.05; **P<0.01.

To investigate the influence of RABEX-5 on migration and invasion, we next performed transwell migration and invasion assays. Downregulation of RABEX-5 suppressed cell migration (SGC-7901/KD group, 121.3±6.1 cells/field; SGC-7901/NC group, 261.0±10.5 cells/field; NCI-N87/KD group, 108.7±6.1 cells/field; NCI-N87/NC group, 241.7±10.6 cells/field; *P*<0.05) (Fig. 4A, C). Similar results were observed in transwell invasion assays (SGC-7901/KD group, 76.3±6.1 cells/field; SGC-7901/NC group, 108.7±6.0 cells/field; NCI-N87/KD group, 60.7±6.0 cells/field; NCI-N87/NC group, 119.0±8.5 cells/field; *P* < 0.05) (Fig. 4B, D).

To investigate the effect of RABEX-5 on motility and wound healing, we also performed wound-healing assays. We observed that the distance between wound edges of SGC-7901/KD and NCI-N87/KD cells was markedly longer than those of SGC-7901/NC or NCI-N87/NC cells (Fig. 4E). In accordance with these observations, levels of VEGF were downregulated at both the mRNA and protein levels in SGC-7901/KD cells (Fig. 4F, G).

### Silencing of RABEX-5 inhibits gastric cancer growth *in vivo*

**Figure 5:**
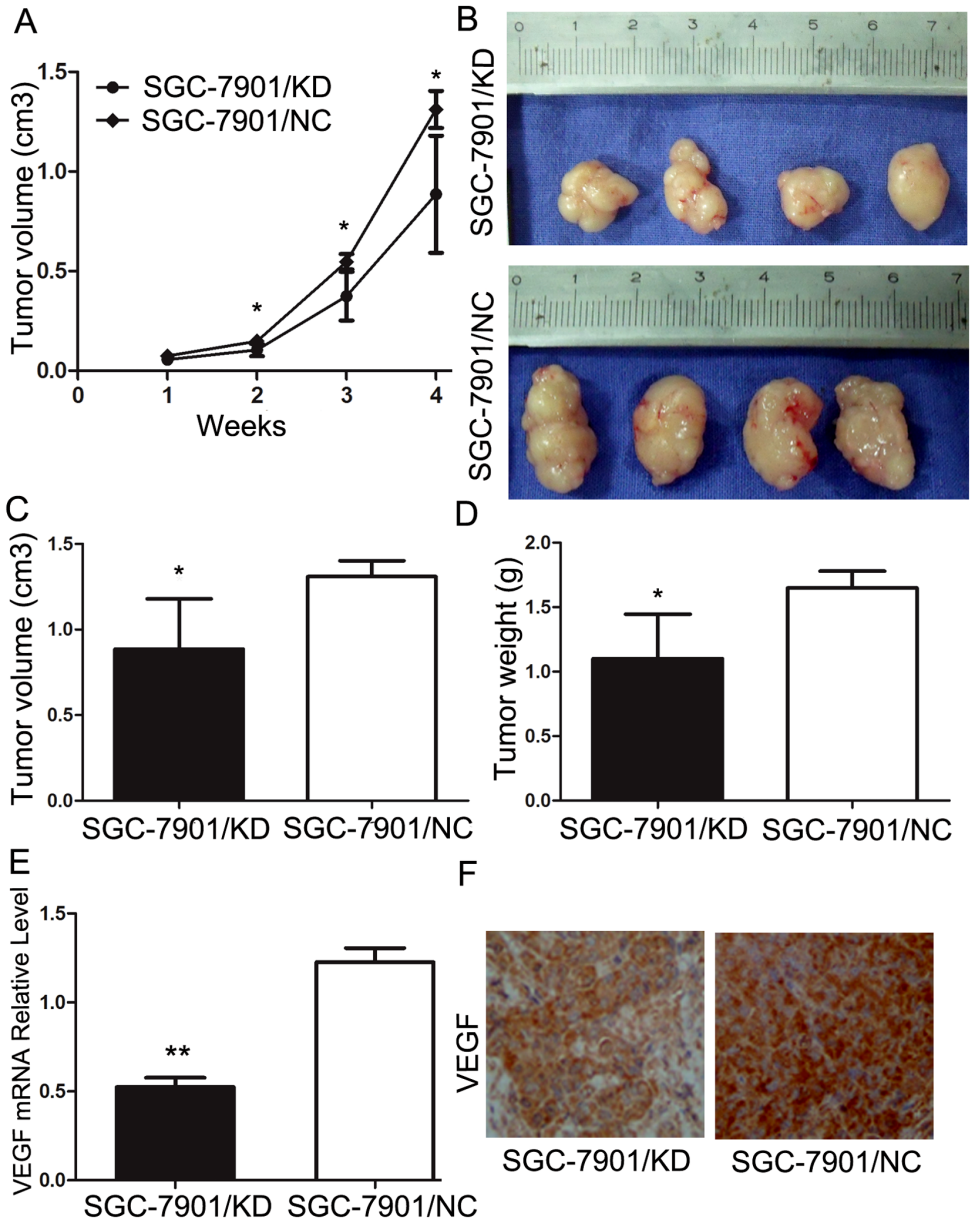
Effect of RABEX-5 on the growth of SGC-7901 cells *in vivo*. (A) Tumor volumes were measured at different time points in mice inoculated with SGC-7901 cells infected with RABEX-5 knockdown lentiviral vector (SGC-7901/KD cells). (B) Tumors harvested from mice 4 weeks post-injection with SGC-7901/KD cells (C) Individual tumor volumes for each mouse after dissection. (D) The final tumor weight was measured at the end of the experiment. (E) Expression of *VEGF* mRNA in tumors derived from nude mice. (F) Immunohistochemical analysis of VEGF expression in tumors derived from SGC-7901/NC and SGC-7901/KD groups. *P<0.05; **P<0.01.

Based on the *in vitro* findings described above, we next examined the effect of RABEX-5 silencing on tumor growth *in vivo* by injecting SGC7901/KD or SGC7901/NC cells subcutaneously into the right flank regions of nude mice. Tumor size was monitored every 3 days with a caliper. Tumor volume was significantly reduced in mice 2 weeks post-injection of SGC-7901/KD cells compared with control cells (*P*<0.05; Fig. 5A). The tumor volume of xenografts derived from the SGC-7901/KD group was comparable to that of the SGC-7901/NC group, exhibiting a marked decrease in tumor volume 4 weeks after tumor cell inoculation (*P*<0.05, Fig. 5B, C). In addition, the weight of tumors derived from SGC-7901/KD cells was significantly less than that of controls (*P*<0.05; Fig. 5D). We next examined the expression of VEGF in transplantation tumor samples by qRT-PCR and immunohistochemistry. As shown in Fig. 5E and 5F, levels of VEGF mRNA and protein were decreased in the SGC-7901/KD group compared with SGC-7901/NC control. These *in vivo* data are consistent with our *in vitro* observations and confirm that silencing of RABEX-5 inhibits gastric cancer growth and progression by modulating VEGF transcriptional activity. In summary, RABEX-5 plays an oncogenic role in gastric cancer.

## Discussion

Although previous studies have shown that RABEX-5 plays a role in tumorigenesis in several types of cancer [11]–[14], its role in gastric cancer had not been investigated. In the present study, we found that RABEX-5 expression is significantly upregulated in gastric cancer tissues compared with adjacent normal tissue, indicating that RABEX-5 may contribute to gastric cancer tumorigenesis. In addition, the expression of RABEX-5 was significantly associated with tumor size and lymph node metastasis. In contrast, there were no significant correlations between abnormal RABEX-5 expression and sex, age or tumor location. This is the first study to elucidate the clinicopathological significance of RABEX-5 expression in patients with gastric cancer.

To investigate the role of RABEX-5 in tumor promotion, we next performed extensive loss of function analysis in RABEX-5 high-expressing cell lines, SGC-7901 and NCI-N87. Successful inhibition of RABEX-5 expression was achieved by RNAi technology, and knockdown was confirmed by western blot. Our analyses revealed that cell proliferation was significantly decreased in SGC-7901/KD and NCI-N87/KD cells and similar results were obtained in colony formation assays. Importantly, *in vivo* xenograft models supported these *in vitro* observations. This phenotype may be caused by RABEX-5 regulating specific genes and signaling pathways, thereby affecting cell cycle and apoptosis. In transwell assays, fewer SGC-7901/KD and NCI-N87/KD cells migrated to the lower chamber than control cells, suggesting that RABEX-5 enhances migration and invasion in gastric cancer cells. Wound healing was also significantly decreased following silencing of RABEX-5. These results verify the important role of RABEX-5 in gastric cancer cell growth and metastatic behavior. However, the exact molecular mechanism by which RABEX-5 exerts these effects remains unknown, since studies involving RABEX-5 are limited.

Previous studies have shown that RABEX-5 can specifically bind to the active form of RAB-5, thereby regulating the docking and fusion of endosomal membranes, the motility of endosomes and intracellular signal transduction [15]. The expression of RAB-5 proteins has been associated with the development of various malignant tumors [16]-[18] and is capable of promoting migration of tumor cells [19], [20]. Angiogenesis plays a key role in tumorigenesis [21], and is regulated by several factors such as the platelet-derived growth factor (PDGF) and the vascular endothelial growth factor (VEGF) [22]. The PI3K/Akt signaling pathway can be regulated by both VEGF and PDGF. For example, VEGF enhances neuroblastoma proliferation by activating PI3K/Akt signaling [23]. Similarly, studies have shown that PDGF can affect the migration ability of cells via the PI3K/AKT pathway [24]. Activation of PI3K/Akt signaling can inhibit cell apoptosis induced by various stimuli [25]–[29], and promote cell cycle progression [30], [31], thereby promoting cell survival and proliferation. This pathway also participates in angiogenesis [32], [33], and plays an important role in tumor formation, invasion and metastasis [34], [35]. Our study shows that RABEX-5 silencing triggers a decrease in VEGF expression. Therefore, we hypothesize that RABEX-5 promotes the growth and metastatic ability of gastric cancer cells through activation of VEGF and its downstream signaling pathways.

In conclusion, we demonstrate that RABEX-5 promotes proliferation, colony formation, migration and invasion, suggesting that RABEX-5 may be an oncogene in gastric cancer, and its effects may be partially mediated by modulation of VEGF activation. RABEX-5 may therefore represent a new diagnostic and prognostic marker and a novel therapeutic target in gastric cancer. More efforts should be made to clarify the signaling pathways underlying these biological phenomena.
